# Molecular and cellular development of spinal cord locomotor circuitry

**DOI:** 10.3389/fnmol.2015.00025

**Published:** 2015-06-16

**Authors:** Daniel C. Lu, Tianyi Niu, William A. Alaynick

**Affiliations:** Department of Neurosurgery, David Geffen School of Medicine, University of California, Los Angeles, Los Angeles, CAUSA

**Keywords:** interneuron, motor neuron, transcription factor, locomotion, sensory, circuit

## Abstract

The spinal cord of vertebrate animals is comprised of intrinsic circuits that are capable of sensing the environment and generating complex motor behaviors. There are two major perspectives for understanding the biology of this complicated structure. The first approaches the spinal cord from the point of view of function and is based on classic and ongoing research in electrophysiology, adult behavior, and spinal cord injury. The second view considers the spinal cord from a developmental perspective and is founded mostly on gene expression and gain-of-function and loss-of-function genetic experiments. Together these studies have uncovered functional classes of neurons and their lineage relationships. In this review, we summarize our knowledge of developmental classes, with an eye toward understanding the functional roles of each group.

## Introduction

**FIGURE 1:**
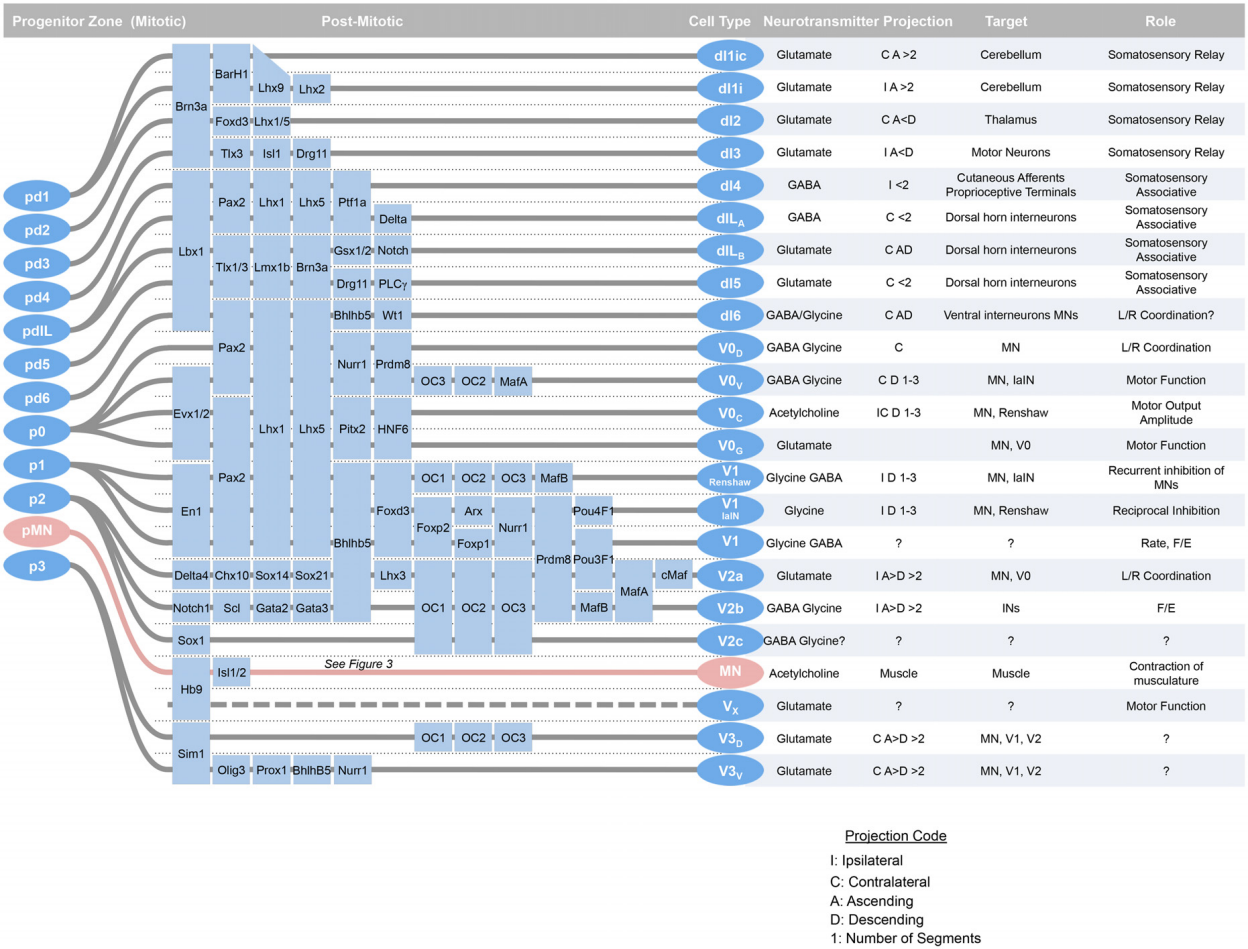
**At around mid-gestation, progenitors exit the cell cycle and begin to take up characteristic setting positions, extend axons, and express transcription factors and neurotransmitter biosynthetic enzymes.** Over the last week of development, 23 classes of neurons can be defined by transcription factor expression. Adapted from Alaynick et al. (2011).

More than 20 distinct embryonic classes of neurons have been described in the spinal cord, and the developmental sources of their diversity have been elucidated over the past decade (**Figure 1**). This cellular diversity has been organized into a schema that defines major groups of neurons based on their expression of embryonic transcription factors. The major characteristics of these classes their generation, transcription factors, subsets, positions, neurotransmitters, connections, and functions are summarized here.

Spinal cord development is subject to phylogenetically ancient organizing principles such as those that guide segmentation from the invertebrates, such as arthropods, to the vertebrates, such as mammals. Cellular identities in vertebrate spinal cord are specified during development along the three basic spatial axes of the embryonic body plan – rostral–caudal, dorsal–ventral, and medial–lateral. In addition, there is a temporal influence of development on these spatial coordinates such that distinct cell fates emerge at different times during development. This yields a four dimensional system for establishing spinal neuron cell fate that has been reviewed extensively (Jessell, 2000; Jankowska, 2001; Lee and Pfaff, 2001; Muroyama et al., 2002; Helms and Johnson, 2003; Goulding and Pfaff, 2005; Kiehn, 2006; Ladle et al., 2007; Stepien and Arber, 2008; Dasen and Jessell, 2009; Goulding, 2009; Grillner and Jessell, 2009; Hegarty et al., 2013).

**FIGURE 2:**
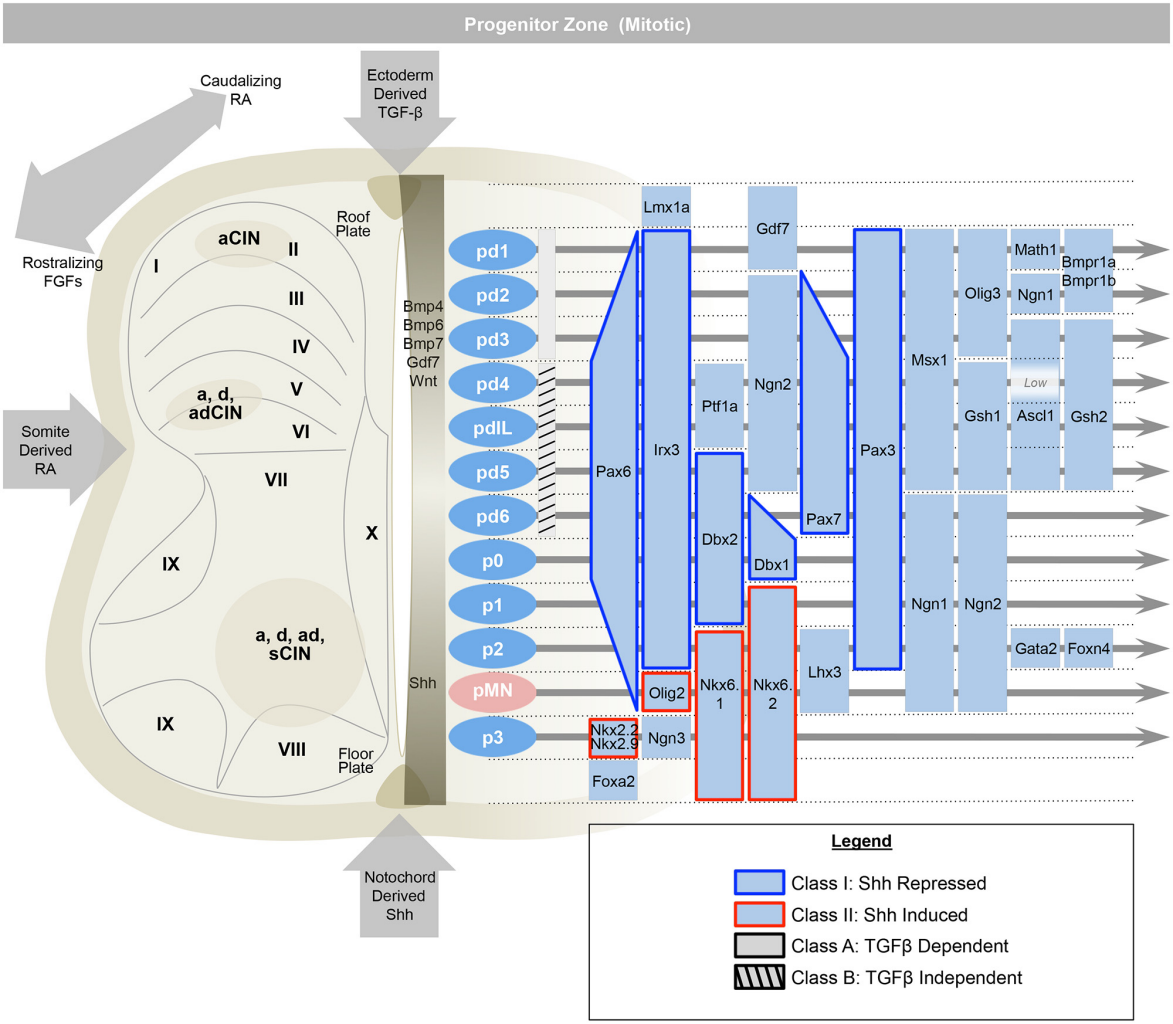
**The early spinal cord (e9.5–e11) is influenced by Sonic-hedgehog (Shh) ventrally, ectoderm-derived TGF-beta family members dorsally, and retinoic acid from the somite, laterally**. This establishes 13 progenitor domains (including the late born pdILA and pdILB) that express transcription factors that help to define progenitor identities and refine boundaries between progenitor domains. Ventrally, Class I transcription factors are repressed by Shh (e.g., *Irx3*), while Class II are induced (e.g., *Olig2*). Similarly, the dorsal-most domains, pd1–pd3, are dependent on TGF-beta and the pd4–pd6 and pdIL domains are independent of TGF-beta signaling. Adapted from Alaynick et al. (2011).

To summarize briefly, the rostral–caudal positional identities are coordinated by opposing gradients of fibroblast growth factor (Fgf, caudalizing) and retinoic acid (RA, rostralizing; **Figure 2**; Muhr et al., 1999; Liu et al., 2001; Dasen et al., 2008). The dorsal–ventral axis is governed by ventralizing Sonic hedgehog (Shh) produced by the floorplate, and dorsalizing signals from the roof plate such as bone morphogenetic proteins (BMPs) and Wnts (which are members of the Wingless + MMTV integrants, *Int* family). These diffusible morphogens form gradients that activate specific transcriptional responses at defined points in the gradient (Roelink et al., 1994; Liem et al., 1995; Ericson et al., 1996; Lee et al., 1998; Megason and McMahon, 2002; Muroyama et al., 2002; Timmer et al., 2002). These transcriptional programs first specify and reinforce the identities of progenitor cells, and second, act to oppose adjacent transcriptional programs and sharpen boundaries between progenitor zones. In the ventral cord, these transcription factors are grouped into two classes, those that are inhibited by Shh (Class I) and those that are activated by Shh (Class II; Briscoe et al., 2000). Spinal cord development is also organized along a medial–lateral axis dividing progenitor cells that are located adjacent to the lumen of the neural tube, medially, whereas differentiating progeny migrate laterally. Over time, a given progenitor domain defined by these spatial coordinates may sequentially produce distinct cellular classes.

Within an idealized spinal cord segment, this system establishes thirteen progenitor pools along the dorsal–ventral axis (**Figure 2**). There are eight dorsal interneuron progenitor divisions, pd1–6 and the late-born pdILA and pdILB, four ventral interneuron progenitor divisions, p0–3, and one motor neuron progenitor domain, pMN (Alaynick et al., 2011). The identities of these domains are predominantly defined by basic-helix-loop-helix (bHLH) domain transcription factors, such as Ngn, Olig2, and Math (Bermingham et al., 2001; Gowan et al., 2001; Novitch et al., 2001; Scardigli et al., 2001; and homeodomain proteins, such as Pax3, Dbx1, and Nkx6.1 (Briscoe et al., 2000; Vallstedt et al., 2001; Subsequently, additional transcription factors, predominantly of the LIM-homeodomain family, such as Lhx1 and Isl1, are expressed in sub-groups of these domains, further refining cell fate into at least 23 distinct classes (Tsuchida et al., 1994; Gross et al., 2002; Muller et al., 2002; Thaler et al., 2002; Cheng et al., 2004).

## Ventral Compartment of Distinct Progenitor Cells

### pMN Fate

**FIGURE 3:**
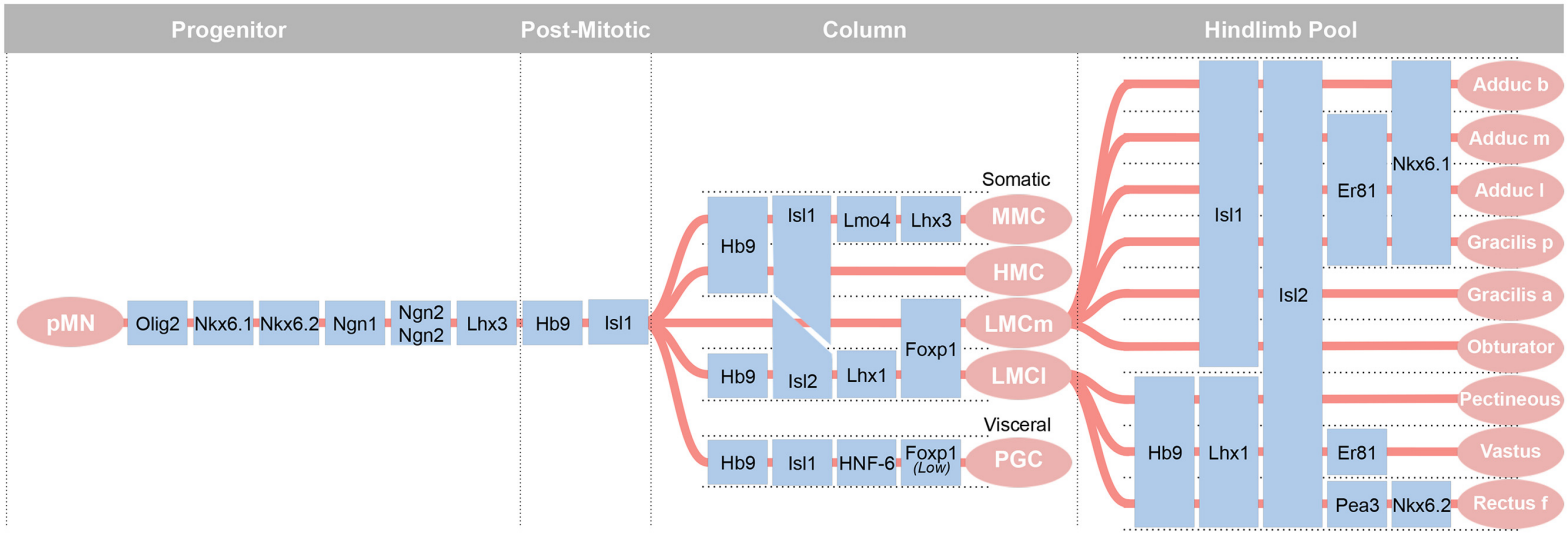
**The most diverse spinal cord neuron class belongs to the motor neurons.** These arise from a uniform progenitor domain before differentiating in to classes that can be grouped by columns and by motor pools. Motor pools are clusters of motor neurons that innervate a single muscle. A transcription factor code is emerging to define each of the over 200 motor pools that innervate distinct muscles. Adapted from Alaynick et al. (2011).

The pMN cell domain gives rise to: (1) 100s of genetically distinct groups of cholinergic alpha motor neurons clustered into motor pools that innervate specific skeletal muscles; (2) gamma motor neurons that innervate intrafusal fibers of specific skeletal muscles for proprioception; (3) the predominantly thoracic (T1–12, and to L1 and L2 in some species) cholinergic preganglionic sympathetic neurons; (4) the cholinergic parasympathetic motor neurons in the sacral (S2–4) cord; and (5) oligodendrocytes found throughout the spinal cord (**Figure 3**). The motor neurons that effect muscle movement are primarily alpha, with fewer beta, motor neurons.

### pMN Birth and Early Development

**FIGURE 4:**
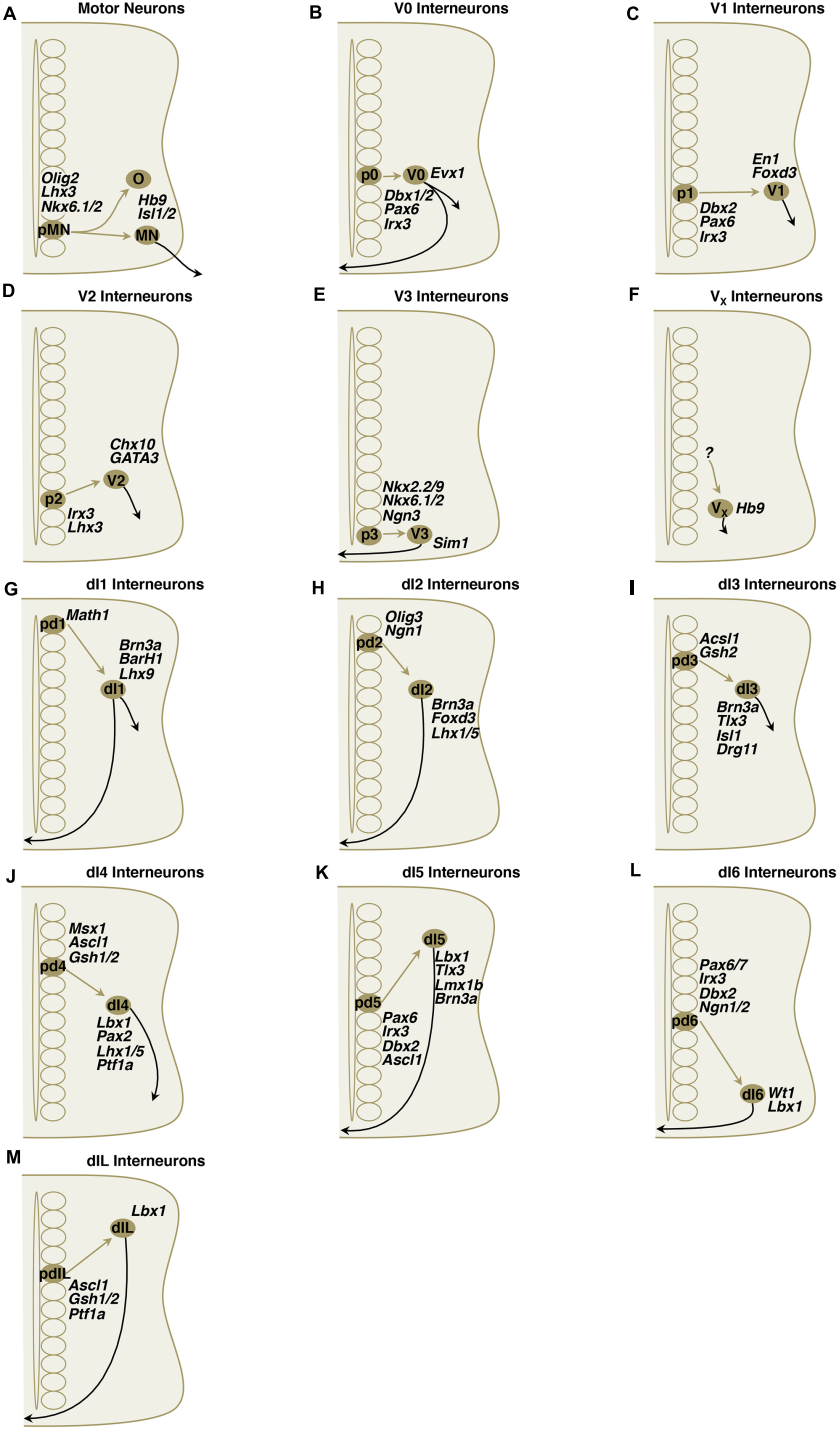
**(A–M)** Simplified schematic illustrations of development of MNs and ventral/dorsal subclass interneurons with important transcriptional factors.

The recognition that a supernumerary notochord could induce the generation of additional motor neurons led to the identification of the diffusible morphogen, Shh, that induces neural precursors to a MN fate (Watterson, 1965; Roelink et al., 1994). An early marker of motor neuron development, the LIM-HD transcription factor, *Isl1*, indicated that motor neuron precursors are born between HH Stages 15 and 17 in chick and beginning at E9.5 (~25 somites) in mouse (**Figure 4A**; Ericson et al., 1992; Roelink et al., 1994; Pfaff et al., 1996; Gould et al., 2006). These progenitors will give rise to somatic alpha motor neurons that innervate skeletal muscle in the medial and lateral motor columns (LMCs), gamma motor neurons that innervate intrafusal fibers of the muscle spindles, and preganglionic motor neurons of the autonomic nervous system. Generation of each of these classes and their organization into motor columns and motor pools requires the subsequent expression of additional transcription factors (Lin et al., 1998; Dasen et al., 2005, 2008). These factors then drive the unique characteristics of that motor pool, such as guidance to the target and establishment of proper connectivity with sensory neurons and interneurons. Interestingly, this transcriptionally defined program is complemented by activity-dependent processes that control cellular connectivity and function (Hanson and Landmesser, 2004; Myers et al., 2005). In the case of gamma motor neurons, the nuclear receptor *Errγ* is expressed in these motor neurons and their survival is dependent on GDNF signaling (Gould et al., 2008; Friese et al., 2009; Shneider et al., 2009; Ashrafi et al., 2012).

The motor neuron progenitor domain is ventral to the *Irx3* expressing p2 domain that delimits *Olig2* expression and is dorsal to the p3 domain that expresses *Nkx2.2* and *Nkx2.9* to delimit *Pax6* expression. The expression of *Nkx6.1* and *Nkx6.2* acts to limit transcription factor expression to *Olig2*, that in turn drives the expression of MN transcription factors *Hb9 (Mnr2 in chick)*, and *Ngn2* (Briscoe et al., 2000; Sander et al., 2000; Vallstedt et al., 2001; Shirasaki and Pfaff, 2002). *Hb9*, expressed during the final cell division of pMNs, is sufficient to drive the expression of *Isl1*, *Isl2*, *Lhx3*, and *ChAT*—as well as its own expression—establishing pMN independence from Shh (Tanabe et al., 1998). Like *Mnr2*, the HD transcription factor, *Hb9*, can induce the formation of motor neurons when ectopically expressed. Loss of *Hb9* in mouse, however, results in ectopic upregulation of a V2 IN marker gene, *Chx10*, but does not result in complete loss of motor neurons or of fictive locomotion (Arber et al., 1999; Thaler et al., 1999; Alaynick, Pfaff unpublished observations).

### Motor Neuron Subtypes

#### The Medial and Hypaxial Motor Columns (MMC and HMC)

The medial sub-group of motor neurons innervates axial musculature and is found the length of the spinal cord. There are two divisions of this group, the medial motor column (MMC) and the hypaxial motor column (HMC or MMCl). Both express *Isl1* and *Isl2*, although the ratio of expression varies, with greater *Isl1* expression in the HMC than MMC at E11.5 and greater *Isl2* in the MMC than HMC by E13.5 in mouse (Tsuchida et al., 1994; Thaler et al., 2004). The MMC innervates dorsal or epaxial musculature, while HMC innervates ventral or hypaxial musculature. Initially all motor neuron progenitors express the LIM homeodomain transcription factor, *Lhx3*. *Lhx3* expression is maintained in the MMC while *Lhx3* expression is downregulated in the HMC and LMC (Tsuchida et al., 1994). Motor neuron (*Hb9* promoter) dependent expression of *Lhx3* results in conversion of LMC motor neurons to a MMC identity (Sharma et al., 2000).

#### The Lateral Motor Column (LMC)

At limb levels, the 50 or so muscles of the limb are innervated by motor neurons occupying a lateral motor column (Landmesser, 1978). Neurons of the lateral portion of the LMC (LMCl) are later born than the MMC motor neurons, and like the cortex, migrate in an inside-out arrangement such that LMC neurons are born in the proliferative ventricular zone of the pMN domain and then migrate through the MMC to form the LMC. While initially expressing *Lhx3*, a hallmark of MMC identity, these motor neurons down-regulate *Lhx3* by an unknown mechanism and begin to express transcription factors not found in MMC that are definitive for LMC identity. The factors include *Foxp1*, *Lim1*, and the enzyme *Raldh2* (Sharma et al., 1998, 2000; Sockanathan and Jessell, 1998). The lateral motor column has lateral (LMCl) and medial (LMCm) divisions that innervate the dorsal and ventral portions of the limb, respectively, and these cell fates are partially regulated by RA signaling (Sockanathan et al., 2003; Ji et al., 2006). In the LMCl, *Lim1* and *Hb9* are expressed while *Isl1* is downregulated. In the LMCm, there is low *Hb9* and maintained *Isl1* expression. The LMCm and LMCl both express *Isl2*, which is downregulated in the MMC and HMC (Misra et al., 2009). The LMCm and LMCl are further subdivided into motor pools, each innervating a specific muscle of the limb. These individual motor pools are defined by their expression of *Ets* and *Nkx* transcription factors that constitute a more refined transcriptional code (De Marco Garcia and Jessell, 2008).

The rostro-caudal regions of the LMC appear to be determined in part by homeobox (Hox) genes. *Hox6* is characteristic of brachial level, *Hox9* of thoracic and *Hox10* of lumbar. Disruption of the Hox genes in mouse or chick has shown that these boundaries can be profoundly altered to create an expansion of lateral motor columns into thoracic regions (Jung et al., 2010). More strikingly, loss of the *Hox* co-factor *Foxp1* disrupts the ability of motor neurons to incorporate the homeobox code for spatial information, and results in a loss of defined motor pools in the LMC (Dasen et al., 2008).

#### Preganglionic Motor Neurons (PGC)

Preganglionic motor neurons of the sympathetic nervous system are the most dorsal motor neurons and can be identified by their expression of *ChAT*, *NADPH Diaphorase*, and some members of the one-cut transcription factor class (Francius and Clotman, 2010). Preganglionic motor neurons are also dependent on the downregulation of *Lhx3* and are lost with continued, *Hb9*-dependent expression of *Lhx3* in all motor neurons, or loss of *Foxp1 or Isl2* (Sharma et al., 2000; Thaler et al., 2004; Dasen et al., 2005, 2008).

## Spinal Interneurons

A great deal has been learned about the development of discrete classes of interneurons by describing them by electrophysiology, behavioral output, and by expression of proteins involved in transcription, neurotransmitter signaling, and intracellular signaling. Currently, this schema has defined over 20 interneuron types in the spinal cord. While one can argue that every neuron has a unique molecular/genetic expression profile, dendritic arborization and axonal projection pattern, this grouping schema has been useful in organizing interneurons into functionally related groups.

Historically, two broad groups have been defined: the “V” interneurons with progenitors that are found in the *v*entral cord and are grossly associated with motor function, and a *d*orsal *I*nterneuron, dI class, associated predominantly with sensory processing. Most studies have examined development within a single or a few segments. A recent study examined rostro-caudal differences at one time point, e12.5 (Francius et al., 2013). This showed that subclasses of ventral interneurons (V0, V1, V2, and V3) exhibit distinct organizational patterns at brachial, thoracic and lumbar levels of the developing spinal cord. Furthermore, each cardinal “V”’ class of ventral interneurons can be subdivided into several subsets according to further combinatorial expression of transcription factors (Francius et al., 2013). Given these caveats that likely apply to other interneuron classes, the V and dI interneuron classifications are a simplification with exceptions, some of which are listed below. Despite these limitations, the V and dI schema is a useful approach to the subject.

### V0 Interneuron Characteristics

**FIGURE 5:**
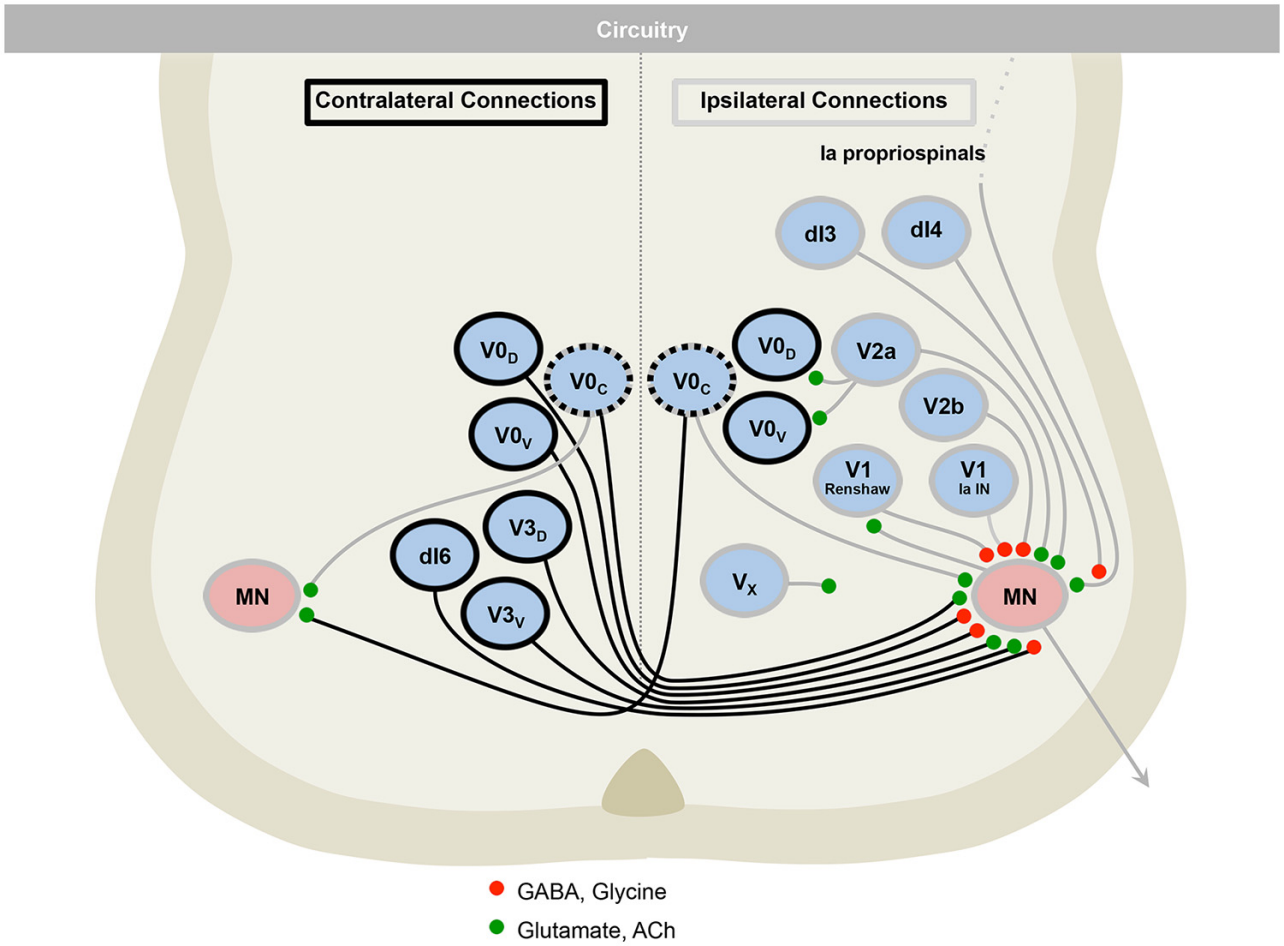
**The motor circuitry is shown in diagrammatic form in the lower panel**. Here, neurons can be divided by projection patterns, that are ipsilateral, contralateral, or both. Three classes of neurotransmitter are found in the cord: excitatory glutamatergic (e.g., V2a), inhibitory GABAergic/glycinergic (e.g., V2b), and excitatory cholinergic neurons (e.g., motor neurons). Roles for neurons in defining rate (e.g., V1), left–right alternation (e.g., V0) and rhythmicity (e.g., V3), are emerging. Adapted from Alaynick et al. (2011).

Local projecting V0 neurons are a population of primarily contralateral, with some ipsilateral projecting neurons with inhibitory or excitatory identity that send axons 2–4 spinal segments rostrally (Moran-Rivard et al., 2001; Pierani et al., 2001). They receive inputs from ipsilaterally projecting Chx10^+^ glutamatergic V2a interneurons (Crone et al., 2008; **Figure 5**). They are the dorsal-most ventral progenitor pool and are characterized by their expression of the *Dbx* (developing brain homeobox) homeodomain transcription factor, *Evx1/2* (even-skipped homeobox 1; **Figure 4B**). *Dbx1* and *Dbx2* are expressed in dividing cells, although *Dbx1* may be briefly expressed in post-mitotic cells (see V1 discussion Pierani et al., 1999). Four V0 interneuron subclasses have been described to date: V0_V_, V0_D_, V0_C_, and V0_G_ (Pierani et al., 1999, 2001; Moran-Rivard et al., 2001; Lanuza et al., 2004; Zagoraiou et al., 2009). Early studies addressed the V0 class by eliminating *Dbx1* and showing that the *Evx1^+^* V0_V_ subclass was lost because these neurons become fated to an *En1^+^* V1-like subclass and astrocytes (Pierani et al., 2001; Lanuza et al., 2004). Because *Dbx1* is transiently expressed, a *Dbx1^LacZ^* knock-in allele was used to show that with loss of *Dbx1*, E18.5 embryos retained 40% of the βgal^+^ cells and resulted in a 25% expansion in the number of *Lbx1^+^ Pax2^+^* dl6-like commissural neurons (Lanuza et al., 2004). By perinatal time points, genetic strategies to track *Dbx1^+^* cells using βgal find that most of these cells are neural by expression of *NeuN* and are found in lamina VIII where commissural interneurons reside. Lineage labeling of Dbx1-derived cell reveals a large abundance of glia (Lanuza et al., 2004). Moreover, the Dbx1 lineage includes many dorsal horn neurons as this transcription factor is also expressed in dorsal domains. Loss of *Dbx1* results in loss of V0_D_ and V0_V_ subclasses, whereas loss of *Evx1* results in a loss of only the V0_V_ subclass (Moran-Rivard et al., 2001; Pierani et al., 2001; Lanuza et al., 2004). V0 and V1 classes both express *Lhx1* and *Lhx5*, markers of inhibitory spinal interneurons (Pillai et al., 2007).

### V0 Birth and Early Development

In mouse, the majority of *Dbx1^+^* progenitors appear between E10 and E13 and give rise to V0_D_ and V0_V_ commissural interneurons (Moran-Rivard et al., 2001; Pierani et al., 2001; Lanuza et al., 2004). *Dbx1/2* expression is found in the rostral CNS at stage 13 in chick and more caudally by stage 15 (Pierani et al., 1999). *Evx1/2* positive V0 cells are generated at stages 17 and 18 and appear in the ventral domain of *Dbx1* and *Dbx2* expression (Pierani et al., 1999). Ventral *Evx1/2* expressing V0 neurons appear at stages 17–18 within the ventral expression domain of *Dbx1* and *Dbx2*, and then migrate ventrally (Pierani et al., 1999). The V0 class appears from a *Pax6^+^, Dbx1/2^+^, Pax3/7^-^* domain that is the dorsal-most ventral progenitor domain (Pierani et al., 1999).

### V0 Interneuron Subtypes

#### V0_V_

The primarily inhibitory V0_V_ class is distinguished by transient expression of the homeodomain transcription factor, *Evx1*. These cells arise from the ventral portion of the *Dbx1^+^* progenitor domain, and like all post-mitotic cells arising from *Dbx1^+^* progenitors, they share a similar post-mitotic migration and commissural axon pattern (Moran-Rivard et al., 2001; Pierani et al., 2001). The V0_V_ interneurons are implicated in locomotion as indicated by increased c-fos immunoreactivity following fictive locomotion (Lanuza et al., 2004). However, *Evx1* knockout mice have grossly normal locomotion patterns despite a ~70% reduction in the V0_V_ interneurons and loss of appropriate contralateral intersegmental axonal projections in the remaining ~30% of interneurons (Moran-Rivard et al., 2001). A subset of the V0_V_ class has been reported to be excitatory in an unpublished observation (Zhang et al., 2008).

#### V0_D_

Unlike the V0_V_ subclass, the more dorsal *Dbx1^+^* progenitors of the glycinergic/GABAergic V0_D_ class do not express *Evx1* (Pierani et al., 2001; Lanuza et al., 2004). And while both V0_D_ and V0_V_ classes have similar axon guidance and cell body position, the loss of the V0_D_ class, in conjunction with V0_V_ class, does appear to alter locomotor behavior. When *Dbx1* is knocked out, eliminating all V0 progenitors, a disruption of left–right coordination is observed at lumbar levels L2 and L5. These periods of left–right synchrony are intermittent and periods of normal left–right alternation are observed amidst episodes of synchrony (Lanuza et al., 2004). No disruption of flexor-extensor behavior, as indicated by alternating phasic activity of the L2 and L5 segments, was observed in a drug-induced isolated cord fictive locomotion assay (Lanuza et al., 2004). Recently, studies have showed that a cluster of V0_D_ cells lateral to the central canal receive substantial input from primary afferents and preferentially project axons toward contralateral motoneurons via an oligosynaptic pathway, and are active during fictive locomotion. This suggests that this subset of V0 interneurons may be primarily responsible for coordination of left–right alternation during locomotion (Griener et al., 2015).

#### V0_C_ and V0_G_

The V0_C_ and V0_G_ subclass represent ~5% of V0 progenitors and are identified by expression of *Pitx2* and occupy a medial position dorsal to the central canal (Zagoraiou et al., 2009). These cells were first observed in lumbar levels at E11.5–12.0 by Pitx2 immunoreactivity which, unlike many embryonic markers, could be detected until postnatal day 30 (Zagoraiou et al., 2009). Neurotransmitter markers can subdivide the *Pitx2^+^* cells into cholinergic (*vAChT^+^* and *ChAT^+^*) and glutamatergic (*vGluT2^+^*) types that are distinct (Zagoraiou et al., 2009). While these are found at cervical and lumbar levels, within the lumbar cord, these two types are distributed in a gradient such that a greater number of cholinergic interneurons are found at more rostral levels and a greater number of glutamatergic interneurons at more caudal levels (Zagoraiou et al., 2009). The cholinergic cells are distinct from *Pitx2^-^* cholinergic C_3_ propriospinal interneurons (Zagoraiou et al., 2009). By genetic tracing, ~80% of these neurons were determined to be from a *Dbx1^+^* progenitor domain at E12.5 and loss of *Dbx1* eliminated the Pitx2 immunoreactivity in the intermediate cord. Because V0_C_ and V0_G_ *Pitx2^+^* cells transiently express *Evx1*, they appear to be subsets of the V0_V_ class. This relatively small ipsilaterally and bilaterally projecting class, however, is responsible for perhaps all c-boutons on motor neurons found in P8 to P25 mice (Zagoraiou et al., 2009; Stepien et al., 2010). These interneurons provide relatively weak innervation to *Sox14::eGFP*^+^ V2a and calbindin^+^ V1 Renshaw cells. These cells appear to be involved in local circuitry as corticospinal and sensory *vGluT1^+^* glutamatergic boutons were not found, whereas serotonergic and *GAD67^+^* GABAergic boutons were observed (Zagoraiou et al., 2009). While previous experiments did not find a gross locomotor behavioral defect with loss of the V0_V_ subclass in *Evx1* mutant animals, *Pitx2* mutant animals were found to have defects in locomotion revealed by EMG recordings during swimming (Zagoraiou et al., 2009). This deficit was argued to represent an abnormal integration of sensory inputs. It may, alternatively or in addition, represent a deficit in C-terminal modulation of motor neuron excitability. A survey in E12.5 mice showed that several V0 subclasses can be defined by expression of *Pax2, Pax6, Evx1, Ptx2, Nurr1, HNF-6, BhlhB5*, and *Prdm8* (Francius et al., 2013).

### V1 Interneuron Characteristics

As a population, this group appears to control burst durations and is comprised of cells physiologists defined as Ia inhibitory and Renshaw cells. Mice models without V1 and V2b showed significant difficulty with limb articulation in flexion and extension (Zhang et al., 2014). The pV1 progenitor domain gives rise to important inhibitory subclasses of neurons that were previously described electrophysiologically: the Ia inhibitory interneurons that mediate reciprocal inhibition and the Renshaw cells that mediate inhibitory feedback to integrate limb and muscle length information into spinal circuitry. Renshaw cells and Ia inhibitory interneurons are V1 derived, but differ in morphology, location, calcium-binding protein expression, synaptic connectivity, and function. These differences are already present in neonates and their differentiation starts in the early embryo (Benito-Gonzalez and Alvarez, 2012). In addition, 75% of V1 interneurons are non-Ia, non-Renshaw subclasses that await characterization (Sapir et al., 2004; Alvarez et al., 2005). Short-range ipsilaterally and rostrally projecting glycinergic/GABAergic V1 neurons are characterized by transient expression of homeodomain transcription factor *En1* (**Figure 4C**; Burrill et al., 1997; Matise and Joyner, 1997; Pierani et al., 1999, 2001; Saueressig et al., 1999). Studies in embryonic chick indicate that these neurons project for only 1–2 segments and have been shown to make inhibitory contacts onto motor neurons and other interneurons, although this may not be the case in the mature mouse (Wenner et al., 1998, 2000). Loss of *Pax6* or *En1*-dependent DTA ablation eliminates the recurrent inhibition by Renshaw cells on motor neurons (Sapir et al., 2004; Gosgnach et al., 2006). Elimination of V1 interneurons results in a marked slowing on the drug-induced fictive locomotion period that is seen in conventional knockouts, targeted ablation, and acute inhibition with allatostatin (Gosgnach et al., 2006; Goulding, 2009). The mechanism by which elimination of an inhibitory class would prolong the locomotor cycle remains unknown and may result from the loss of inhibitory neurons to terminate MN firing. The V1 class, like V0, expresses the inhibitory spinal interneuron markers *Lhx1* and *Lhx5* (Pillai et al., 2007).

### V1 Birth and Early Development

Unlike cells in the dorsal-most p0 domain that expresses *Dbx1* and *Dbx2*, the adjacent p1 domain only expresses *Dbx2* (Pierani et al., 1999). The V1 class appears from a *Pax6^+^, Dbx2^+^, Nkx6.2^+^, Dbx1^-^* domain that is ventral to the *Dbx1/2^+^* V0 domain (Matise and Joyner, 1997; Pierani et al., 1999). In chick, *En1^+^, Lim1/2^+^* V1 neurons appear at stage 17, and most appear ventral to the domain of *Dbx1* expression, within the ventral domain of these *Dbx2^+^, Dbx1^-^* progenitors (Pierani et al., 1999). *Dbx* expression does not overlap with *En1*, perhaps due to the relatively late expression of *En1* (Pierani et al., 1999). Genetic tracing studies using *Dbx1^nlsLacZ^* mice between ages E10 and E16.5 found that ~5–10% of *En1*^+^ cells did express a low level of βgal, perhaps a reflection of transient *Dbx1* expression and more enduring βgal protein. The V1 class is marked by expression of *Foxd3*, found in the dI2 domain, as well (Ramos et al., 2010). The transcription factor, *Bhlhb5*, which marks the V1, V2 and dI6 domains, is required at least partially for V1 identity assessed by *En1* expression (Ramos et al., 2010; Skaggs et al., 2011). Expression of *Bhlhb5* in conjunction with *Ngn2* facilitates V1 identity ectopically (Skaggs et al., 2011).

### V1 Interneuron Subtypes

#### V1 Renshaw

Renshaw cells use both glycine and GABA as neurotransmitters, transiently express *Gad65* early in embryonic development and have both motor neurons and Ia interneurons as targets (Saueressig et al., 1999; Sapir et al., 2004). They also express calbindin D28K embryonically and continue to express this marker into adulthood (Alvarez et al., 1999; Geiman et al., 2000). They receive input from motor neuron collaterals that release acetylcholine, glutamate, and aspartate (Mentis et al., 2005; Richards et al., 2014). Renshaw cells modulate proprioceptive sensory input and motor neuron output. Genetic tracing studies showed that Renshaw cells are derived from an *En1*^+^ progenitor pool and, although they are not lost in the absence of *En1*, they do have fewer motor neuron recurrent inputs (Sapir et al., 2004). They are, however, lost in the absence of *Pax6* (Sapir et al., 2004). Recent study showed that selective activation of the Onecut transcription factors Oc1 and Oc2 during the first wave of V1 interneuron neurogenesis is a key step in the Renshaw cell differentiation; furthermore Renshaw cell development is dependent on the forkhead transcription factor Foxd3, which is more broadly expressed in post-mitotic V1 interneurons (Stam et al., 2012).

#### V1 Ia Interneuron

Although Ia interneurons have been rediscovered as a V1 subclass, like Renshaw cells, the Ia INs were functionally described before the advent of molecular genetic dissection of interneuron development (Eccles et al., 1954; Hultborn et al., 1971; Hultborn and Udo, 1972) These inhibitory glycinergic cells receive input from muscle spindle Ia proprioceptive afferents carrying muscle length information and provide inhibitory input onto motor neurons innervating antagonist muscles. Like motor neurons, Ia receive inhibitory inputs from Renshaw cells (Hultborn et al., 1971). In neonatal mice, disynaptic glycinergic reciprocal inhibition is mediated by Ia interneurons, although this activity is preserved in the absence of *Pax6*, indicating that cells of more than one origin contributes to this functional class (Wang et al., 2008). Only when V1 and V2b are both ablated is reciprocal inhibition profoundly altered. Renshaw cells constitute 8–19% of V1 interneurons and the *Foxp2*^+^ (by immunohistochemistry) population accounts for around 33% of these neurons at P0 and 50% at E13 (Morikawa et al., 2009). Because there are no universal markers of Ia interneurons, all Ia interneurons cannot be accounted for, leaving the physiologic properties and connectivity patterns of V1 interneurons unaccounted for (Alvarez et al., 2005). Of note, some interneurons with synaptic organization like Ia interneurons have been found that arise from the V1 population and are Foxp2 positive (Morikawa et al., 2009). A survey in E12.5 mice showed that several V1 subclasses can be defined by expression of *Calbindin, OC1, OC2, OC3, Foxd3, En1, MafB, FoxP2, Foxd3, Foxp4, Pax2, Arx, Evx1, Nurr1, BhlhB5, Pou4F1, Pou3F1*, and *Prdm8* (Francius et al., 2013).

### V2 Interneuron Characteristics

V2 interneurons become divided into V2a and V2b classes of ipsilaterally projecting interneurons that extend axons caudally across several segments (Goulding, 2009). The excitatory V2a class is glutamatergic and expresses *Chx10*, while the *Gata2/3*^+^ V2b class is inhibitory and uses both glycine and GABA (**Figure 4D**; Al-Mosawie et al., 2007; Lundfald et al., 2007). The transcription factor, *Bhlhb5*, marks the V2, as well as V1 and dI6 domains (Ramos et al., 2010).

### V2 Birth and Early Development (Notch-Delta)

V2 interneurons arise from a progenitor pool just dorsal to the pMN domain and share expression of *Lhx3* with the pMN domain. In addition, both domains share expression of NLI that forms homodimers. This NLI homodimer nucleates the formation of a higher-order tetramer with Lhx3 in the V2 progenitor domain, and in the case of pMNs this V2-defining tetramer (Lhx3-NLI-NLI-Lhx3) is disrupted by the insertion of Isl1 to form a hexamer (Lhx3-Isl1-NLI-NLI-Isl1-Lhx3). Transcriptional response elements that are active in V2 cells can bind both the motor neuron hexamers and the V2 associated tetramers, while response elements active in motor neurons are only responsive to the hexamers (Lee et al., 2008). Later the V2 domain expresses *Chx10* that acts as a repressor of motor neuron associated hexamers in V2 progenitors, leaving only the LIM tetramers active (Sander et al., 2000; Lee et al., 2008). The progenitor pool of V2 neurons becomes post-mitotically segregated into V2a and V2b neurons.

Time-lapse imaging in zebrafish showed that the majority of V2 progenitors give rise to a pair of V2a and V2b cells (Kimura et al., 2008), indicating that V2a and V2b arise from the same progenitor. This segregation into V2a and V2b is mediated by Notch/delta signaling in zebrafish and mouse models (Yang et al., 2006; Del Barrio et al., 2007; Peng et al., 2007). In mouse, Delta4, but not Delta 1, activates this signaling cascade and is downstream of *Foxn4*, which also induces expression of *Mash1/Ascl1* (Del Barrio et al., 2007; Peng et al., 2007). Mind bomb-1 (Mib1) is an E3 ubiquitin ligase that ubiquitinates and promotes the endocytosis of Notch ligands. In mice model, Mib1 plays an important role in Notch activity and specific differentiation, neurogenesis and gliogenesis of V2 interneurons. Mice models with abnormal Mib1 resulted in unclear spinal progenitors, premature or unbalanced differentiation or loss of astrocytes and oligodendrocytes (Kang et al., 2013). In zebrafish embryos two ligands, DeltaA and DeltaD, and three receptors, Notch1a, Notch1b, and Notch3 redundantly contribute to p2 progenitor maintenance; on the other hand, DeltaA, DeltaC, and Notch1a mainly contribute to the V2a/V2b cell fate determination (Okigawa et al., 2014). Misra et al. (2009) showed Foxn4 and proneural factors may serve as the trigger to initiate asymmetric Dll4-Notch and subsequent BMP/TGFβ signaling events required for neuronal diversity in the V2 domain (Okigawa et al., 2014). V2b fate is specified by active *Notch1, Foxn4, Mash1*, and *Scl* Notch-binding protein MAML is also required for this specification (Peng et al., 2007). Lack of active Notch1 results in V2a fate, shown in an increase of V2a interneurons at the expense of V2b in *Psn1* KO mice or *Notch1* KO mice (Del Barrio et al., 2007; Peng et al., 2007). Transcription factor Gata2 is necessary in the normal development of V2a and V2b neurons and Gata2 promotes the selective activation of V2b at the expense of V2a fate (Francius et al., 2014). Progenitors that express the notch ligand, Delta-like 4 generate almost all V2a and V2c neurons while producing only a small fraction of neurons of other subtypes along the dorsoventral axis (Zou et al., 2015).

### V2 Interneuron Subtypes

#### V2a Sox14/Chx10

The V2a class of ipsilaterally projecting interneurons expresses the transcription factors Chx10 and Sox14 and is glutamatergic. These interneurons are composed of cells with diverse firing properties and morphologies with local as well as long-range ipsilateral projection patterns (Dougherty and Kiehn, 2010a,b; Zhong et al., 2010). This class of interneurons has been shown to contact motor neurons (Al-Mosawie et al., 2007; Stepien et al., 2010) and contralaterally projecting V0 interneurons (Crone et al., 2008). Loss of these cells has been shown to disturb locomotor function in a state-dependent manner (Crone et al., 2008, 2009; Dougherty and Kiehn, 2010a,b; Zhong et al., 2010). In a series of *in vitro* experiments it was found that *Chx10-DTA* V2a-ablated mice displayed more variable amplitude and period than wild-type controls during drug-induced fictive locomotion. Further, these mutant animals had incoherent left–right alternation during drug-induced fictive locomotion. Surprisingly, these animals failed to display coordinated brainstem stimulated or dorsal root stimulated fictive locomotion, suggesting that *Chx10*^+^ cells mediate descending and sensory activation of locomotor activity (Crone et al., 2008).

A subsequent study, using a different strain of mice that avoided the neonatal lethality seen in previous work, showed that during treadmill running, *Chx10-DTA* mice can transition from alternating locomotion to synchronous hindlimb locomotion at higher speeds. High-speed synchronous left–right activity, or galloping, is not normally seen in mice, although it has been described in studies of *Eph* and *ephrin* signaling molecule mutant mice (Dottori et al., 1998; Kullander et al., 2001; Yokoyama et al., 2001). The *Eph/ephrin* mutant mice, however, have synchronous activity at both slow and fast speeds. Some V2a interneurons express *EphA4*, but a compelling correlation has yet to be discovered (Lundfald et al., 2007). In zebrafish, *alx*, a zebrafish homolog of *Chx10*, is expressed in an ipsilateral descending excitatory interneuron population named CiD (circumferential descending) neurons that monosynaptically contact motor neurons (Kimura et al., 2006; McLean et al., 2008). This population has been shown to be active during high-frequency swimming in larval zebrafish (McLean et al., 2008). Within this interneuron class, dorsally located cells are recruited at a high swimming frequency. As the frequency decreases, more ventral cells are recruited, accompanied by silencing of previously active dorsal cells (McLean et al., 2008). A survey in E12.5 mice showed that V2a subclasses can be defined by expression of *BhlhB5, Pou3F1, OC1, OC2, OC3, Prdm8, MafA*, and *cMaf* (Francius et al., 2013).

#### V2b Gata2/3

Ipsilaterally projecting V2b interneurons express *Gata2/3*, are inhibitory GABAergic neurons, and appear to make direct connections onto motor neurons (Lundfald et al., 2007; Peng et al., 2007). Observations by the Goulding lab indicate they project caudally (Zhang et al., 2014). These cells may underlie the retained reciprocal inhibitory pathways seen in V1 knockout mice (Wang et al., 2008). A survey in E12.5 mice showed that V2b subclasses can be defined by expression of *BhlhB5, Pou3F1, OC1, OC2, OC3, Prdm8, MafA*, and *MafB* (Francius et al., 2013). As pointed out earlier, V1- and V2b-derived neurons function as the core interneuronal components of the limb central pattern generator (CPG) that coordinate flexor-extensor motor activity (Zhang et al., 2014).

#### V2c Sox1

The V2 interneuron class has recently been shown to further diverge to a Sox1-expressing Gata3-negative population named V2c interneurons, function of which is still yet to be elucidated (Li et al., 2010; Panayi et al., 2010). A survey in E12.5 mice showed that V2c subclasses can be defined by expression of *Sox1, OC1, OC2, and OC3* (Francius et al., 2013).

### V3 Interneuron Characteristics

The *Sim1*^+^ *VGluT2*^+^ glutamatergic V3 interneurons send projections predominantly contralaterally and caudally (Goulding, 2009). Genetic tracing, using a *Sim1-eGFP* or *Sim1^Cre^* and reporter lines, and viral tracing, using pseudorabies, shows that 80–85% of these cells project contralaterally and a minor proportion remain ipsilateral or project both contra- and ipsilaterally (Zhang et al., 2008). As a population, *Sim1*^+^ V3 interneurons form 24% of glutamatergic connections on V1 Ia, 27% on Renshaw subclasses, 22% of glutamatergic synapses on lateral motor column motor neurons, as well as connections on *Lhx3*^+^ V2 interneurons, and lamina VIII commissural interneurons (Zhang et al., 2008). Behaviorally, loss of V3 neuronal activity by genetic attenuation with tetanus toxin or allatostatin signaling resulted in a loss of CPG robustness. In isolated cord fictive locomotion, both dorsal root stimulation and drug-induced methods produced weak CPG activity in only some of the cords examined. The outputs were less consistent and had greater coefficients of variance. Although both right and left sides of the cord produced irregular outputs, the fidelity of left–right coordination was preserved suggesting that V3 interneurons do not regulate the coordination of left–right activity. In adult *Sim1^Cre^ AlstR192* animals, application of allatostatin to the cord produced locomotor disturbances in gait, as well (Zhang et al., 2008). In Sim1 mutant mice, V3 interneurons are produced normally and maintain in the similar position and organizations as wild-type; however, there is significant reduction of interneurons in dorsal subgroup and there is significant reduction in the contralateral axonal projection. Therefore, Sim1 appears to be critical in migration and axonal projection of V3 interneuronal development (Blacklaws et al., 2015). Mice that are mutant for *Nkx2.2* and *Nkx2.9* lose V3 interneurons and *Nkx2.2^+/-^ Nkx^-/-^* mice display intermittent or permanent hopping gait (Holz et al., 2010). Holz et al. (2010) indicate that this mutation affects floor plate, and therefore likely affects commissural interneuron projections that mediate left–right coordination. A survey in E12.5 mice showed that V2c subclasses can be defined by expression of *Olig3, Prox1, BhlhB5*, and *Nurr1* (Francius et al., 2013).

### V3 Birth and Early Development

These V3 interneurons arise from the ventral-most p3 progenitor domain defined by homeobox transcription factors *Nkx2.2* and *Nkx2.9* and the PAS-bHLH transcription factor *Sim1* (simple-minded homolog 1; **Figure 4E**; Briscoe et al., 1999; Goulding et al., 2002). Genetic tracing techniques using a *Sim1^TauLacZ^* knock-in reporter mouse or *Sim1^Cre^* and reporter lines (*R26^floxstop-GAP43-GFP^* and *R26^floxstop-lacZ^*) have shown similar expression at E11.5 to *in situ* hybridization data for *Sim1* expression that appeared just lateral to the *Nkx2.2* progenitors (Marion et al., 2005; Zhang et al., 2008). *Nkx2.2* also regulates the expression of *Olig3* in V3 neurons. While *Olig3* plays a key role in respecification of dl2 and dl3 neurons into dl4 interneurons in dorsal spinal cord (see below), it does not appear to affect the generation and migration of the ventral neurons (Liu et al., 2014).

#### V3_**D**_ and V3_**V**_

Each class of interneurons can likely be further subdivided. The existence of V3 subtype heterogeneity defined by cell body positions was first reported in a review of locomotor circuitry by the Goulding group (Goulding, 2009). This group recently examined both electrophysiological and morphological properties of mature V3 interneurons in adult mouse and were able to identify two V3 subpopulations with distinct intrinsic properties and distributions (ventral and dorsal), as well as an important intermediate subgroup (Borowska et al., 2013). They reported V3_V_, primarily located in lamina VIII, possessed a few branching processes and were capable of generating rapid tonic firing spikes and V3_D_ had a more complex morphology with relatively slow average spike frequency with strong adaptation (Borowska et al., 2013). A survey in E12.5 mice showed that V3_V_ express *Olig3, Prox1, BhlhB5*, and *Nurr1*, and V3_D_ can be defined by expression of *OC1, OC2*, and *OC3* (Francius et al., 2013).

#### V_X_ Hb9

A group of glutamatergic, rhythmically active interneurons with possible connections to motor neurons can be found along either side of the ventral midline in thoracic and upper lumbar segments (Thaler et al., 1999; Wichterle et al., 2002; Hinckley et al., 2005; Wilson et al., 2005). These *Hb9*^+^ and *VGluT2*^+^ interneurons are found in lamina VIII, although the developmental origin of these cells is unknown (**Figure 4F**). These cells have oscillatory behavior, make potential contacts with motor neurons, and are associated with motor rhythms (Hinckley et al., 2005; Wilson et al., 2005; Hinckley and Ziskind-Conhaim, 2006). These interneurons were the first to show oscillatory properties and efforts have been made to discover a relationship to rhythm generation or a pacemaker property for the CPG (Kwan et al., 2009). No cell class, however, has been found to act as a pacemaker for CPG activity. Remaining questions for the V_X_ include: what is the progenitor domain that gives rise to the V_X_ domain; and why are they not found below the L2 segment at E18.5.

## Dorsal Interneuron Progenitors

There are eight canonical classes of dorsal progenitors, dI1–6 and dIL_A_ and dIL_B_. Of these, the dorsal-most dI1–3 progenitors are dependent on signals from the roof plate and termed Class A (Liem et al., 1997; Lee et al., 2000). The remaining dI4–6 and dIL_A_ and dIL_B_ are independent of roof plate signals and termed Class B (Gross et al., 2002; Muller et al., 2002). The dorsal-most progenitors, pd1–pd3, are born between days E9.5 and 10.5 and become post-mitotic and begin to migrate ventrally between E10.5 and E11.5 (Helms and Johnson, 1998; Bermingham et al., 2001; Gross et al., 2002; Muller et al., 2002). These cells will eventually form the deeper layers of the dorsal horn. The more ventral Class B dI4–6 cells are born between E10 and 12.5 and then post-mitotically express *Lbx1* and migrate either dorsally to form the more superficial layers of the dorsal horn or migrate ventrally to the deep dorsal horn and the ventral spinal cord (Gross et al., 2002; Muller et al., 2002). The later born dIL_A_ and dIL_B_ classes are born between E11 and E13 and are inter-mixed with each other. They then migrate dorsally and constitute a significant portion of the cells in the superficial dorsal horn, including the substantia gelatinosa (Nornes and Carry, 1978; Gross et al., 2002; Muller et al., 2002; Mizuguchi et al., 2006). As with the ventral interneuron classes, each of these classes, or their subgroups, has characteristic features. For instance, each interneuron subclass appears to have a unique axonal projection that produces a tight fascicle within white matter tracts (Avraham et al., 2009, 2010).

### dI1 Interneuron Characteristics

The dorsal-most progenitor domain pd1 expresses the bHLH transcription factor *Math1*^+^ (Mouse atonal homolog 1, also known as *Atoh1*) and gives rise to at least two *VGluT2*^+^ glutamatergic subclasses: dI1A and dI1B, characterized by Lim-HD expression and their spinocerebellar tract (SCT) contributions (**Figure 4G**). Recent study shows that *Msx1* and *Msx2*, two homeodomain transcription factors that are induced earlier than bHLH transcription factors, likely play a role as transcriptional activators of *Math1*/*Atoh1* in spinal cord development (Duval et al., 2014). The dI1A (also known as dI1_comm_) neurons express the Lim-HD transcription factors *Lhx2_high_* and *Lhx9_low_*, while dI1B (also known as dI1_ipsi_) express the Lim-HD TF *Lhx9* (Helms and Johnson, 1998; Lee et al., 1998; Bermingham et al., 2001; Gowan et al., 2001; Wilson et al., 2008; Avraham et al., 2009). The dI1 interneurons migrate to the deep dorsal horn and intermediate gray where they receive proprioceptive input from the periphery and form commissural projections of dorsal and ventral SCTs (Helms and Johnson, 1998; Bermingham et al., 2001). Using an *Atoh1^LacZ^* allele to trace the fate of pd1 progenitors in developing mouse, at least two subsets of the dI1 class have been identified: (1) a medial cluster of vertically oriented neurons that are *Cbln2*^+^ and *Smarca2*^+^ and projects to the SCT in the contralateral (*Tag1*^+^) lateral funiculus; (2) a more lateral *Sox6*^+^, cluster of horizontally oriented neurons that contributes to the SCT in the ipsilateral lateral funiculus (Miesegaes et al., 2009). In chick, data with an enhancer that labels these cells suggests that both fascicles coalesce in the lateral funiculus ventral to the fascicle formed by the dI2 projections (Avraham et al., 2009).

### dI1 Birth and Early Development

The roof-plate-dependent Class A dp1 progenitors of the dI1 class express the bHLH transcription factors *Olig3* and *Math1* (Muller et al., 2005; Gowan et al., 2001). The dI1 neurons in mouse are born between E10 and E12.5 and express *Lhx2/9*, *Barhl1* (bar homeobox like 1) and *Brn3a* (*Pou4f1*, a class IV POU domain-containing transcription factor; Helms and Johnson, 1998). Loss of function experiments with BMP7 in chick and *Bmp7* mutant mice results in loss of dI1, dI3, and dI5 (Le Dréau et al., 2012).

### dI2 Interneuron Characteristics

dI2 interneurons are ascending, contralaterally projecting, relay interneurons that migrate to the intermediate spinal cord and ventral horn (Gowan et al., 2001; Gross et al., 2002). These interneurons have been suggested to convey sensory information via the spinothalamic tract to the thalamus, based on their location (**Figure 4E**; Brown, 1981; Tracey, 1985; Gross et al., 2002). The projections likely occupy the lateral funiculus and are dorsal to the dI1 fascicle, as analyzed by enhancer expression in chick (Avraham et al., 2009). Arising from bHLH transcription factor *Ngn1* (neurogenin 1) and *Ngn2* expressing progenitors, these neurons express LIM-HD transcription factors *Lhx1*, *Lhx5* and winged-helix domain *Foxd3* (forkhead homeobox D3) post-mitotically (Bermingham et al., 2001; Gowan et al., 2001; Gross et al., 2002). These interneurons were previously known as D3A interneurons.

### dI2 Birth and Early Development

The roof-plate-dependent Class A dI2 progenitor domain, pd2, is characterized by the expression of the bHLH transcription factors, *Olig3*, *Ngn1*, and *Ngn2* and are born between E10- and E12.5 (**Figure 4H**; Gowan et al., 2001; Muller et al., 2005). Two SoxD transcription factors, Sox5 and Sox6, are expressed in restricted domains of dorsal progenitors. Sox5 controls cell fate specification of dp2 and dp3 progenitors and, as a result, controls the correct number of the corresponding dorsal interneurons (dI2 and dI3; Quiroga et al., 2015).

### dI3 Interneuron Characteristics

The dI3 neurons are excitatory interneurons in the deep dorsal horn and intermediate spinal cord (Liem et al., 1997; Gowan et al., 2001; Cheng et al., 2004). These cells target motor neurons monosynaptically, as revealed by recent rabies tracing experiments (Stepien et al., 2010). They have axons that project rostrally, ipsilaterally, and longitudinally in two fascicles. A ventral fascicle enters the ventral lateral funiculus (VLF) and the dorsal fascicle enters the dorsal funiculus (DF; Avraham et al., 2010). The dorsal projecting axons re-enter the cord when they encounter axons sensory axons at the dorsal root entry zone (DREZ; Avraham et al., 2010). Similarly, the ventrally projecting neurons re-enter the cord at ventral root exit points (Avraham et al., 2010). In mice model, dl3 appears to convey input from low threshold cutaneous afferents to the motor neurons that is critical in hand/forelimb grip (Bui et al., 2013). The dI3 pool also expresses *Tlx3* (T-cell leukemia homeobox 3) and LIM-HD transcription factor *Isl1*-expressing cells (**Figure 4I**; Gross et al., 2002). The turning behavior of dI3 neurons is dependent on *Isl1*, and expression of *Isl1* in dI1 neurons conferred dI3-like axon choice points to dI1 neurons (Avraham et al., 2010). *Tlx1* (also known as *Hox11*) and *Tlx3* (also known as *Rnx* and *Hox11L2*) are markers of glutamatergic signaling. *Tlx3* functions cell-autonomously to specify a glutamatergic neurotransmitter phenotype (Cheng et al., 2004).

### dI3 Birth and Early Development

The roof-plate dependent Class A dP3 progenitors express the basic helix-loop-helix (bHLH) transcription factor *Mash1* (*Ascl1*, Mouse Achaete-scute complex-like 1), as do adjacent pd4 and pd5 domains (Gowan et al., 2001; Helms et al., 2005). They also express *Olig3*, *Pax7* and *Ngn2* and *Gsh2* (Muller et al., 2005). In chick spinal cord electroporation experiments it has been shown that over-expression of *Olig3* increases dI3 interneurons at the expense of other Classes A and B neuron classes and this effect is enhanced by *Mash1* (Muller et al., 2005). Over-expression of *Mash1* results in more dI3 and dI5 neurons at the expense of dI2 and dI4 (Muller et al., 2005), while loss of Mash1 causes a decrease in dI3 and dI5 populations while dI4 is maintained (Helms et al., 2005). As mentioned above, loss of function experiments with BMP7 in chick and *Bmp7* mutant mice results in loss of dI1, dI3, and dI5 (Le Dréau et al., 2012).

### dI4 Interneuron Characteristics

The early born (E10.5–E11) dI4 interneurons become *Pax2*^+^, *Lhx1*^+^, and *Lhx5*^+^ GABAergic ipsilaterally projecting somatosensory associative neurons that migrate laterally to the deep dorsal horn (**Figure 4J**; Gross et al., 2002; Muller et al., 2002; Pillai et al., 2007). In addition, both dI4 and dI5 interneurons also express *Gsh1* (*Gsx1*) and *Gsh2* post-mitotically, while dI3 only express *Gsh2* (Kriks et al., 2005; Muller et al., 2005; Mizuguchi et al., 2006). They are GABAergic, calbindin^+^ and express the nociceptive marker *PLCγ* (Chen et al., 2001; Helms and Johnson, 2003). The dI4 fate is dependent on *Ptf1a* and loss of this gene results in loss of all GABAergic dorsal neurons and respecification to dI5 fate (Henke et al., 2009; Meredith et al., 2009). Loss of *Lhx1* and *Lhx5* results in a loss of *Pax2*, *Viaat*, and *Gad1* (Pillai et al., 2007). In addition, *Pax2* is required for the maintained expression of *Lhx1, Lhx5, Pax5*, and *Pax8* (Pillai et al., 2007).

### dI4 Birth and Early Development

The roof-plate independent Class B dP4 domain expresses *Lbx1*, *Mash1* and higher levels of *Pax7* than the dorsally adjacent pD3 domain (Gross et al., 2002; Muller et al., 2002). These progenitors are born between E10.5 and E11 and this domain is distinct from the dIL progenitor domain that produces dIL_A_ and dIL_B_ progenitors, although both have very similar transcription factor expression patterns (below). *Olig3* over-expression can inhibit formation of dI4, and loss can result in expansion of this domain (Muller et al., 2005). While these cells express *Mash1*, loss of *Mash1* does not block dI4 formation, yet it does disrupt dI3 and dI5 (Helms et al., 2005).

### dI5 Interneuron Characteristics

The roof-plate independent Class B dI5 neurons become contralaterally projecting glutamatergic somatosensory interneurons of the deep dorsal horn (nucleus proprius) and ventral horn that express the homeodomain transcription factors *Lbx1*, *Brn3a*, *Tlx1*, *Tlx3*, and *Lmx1b* (**Figure 4K**; Gross et al., 2002; Muller et al., 2002; Qian et al., 2002; Ding et al., 2005; Glasgow et al., 2005). In addition, a subset expresses *PhoxA2* (Ding et al., 2004). These interneurons were previously known as D4.

### dI5 Birth and Early Development

These cells are born between E10.5–E11 and arise from a *Mash1*^+^ and *Pax7*^+^ dP5 progenitor domain that express *Lbx1* post-mitotically to both reinforce Class B fate and oppose Class A fates (**Figure 4K**; Gross et al., 2002; Muller et al., 2002). The dI5 domain expresses *Gsh1* and *Gsh2*, as does the adjacent dI4 domain (Kriks et al., 2005). As noted previously, loss of function experiments with BMP7 in chick and *Bmp7* mutant mice results in loss of dI1, dI3, and dI5 (Le Dréau et al., 2012).

### dI6 Interneuron Characteristics

The roof-plate independent Class B dI6 commissural inhibitory interneurons express *Lbx1*, *Lhx1*, *Lhx5*, and are *Pax2* positive, indicating a GABAergic fate (**Figure 4L**; Gross et al., 2002; Muller et al., 2002; Cheng et al., 2004; Glasgow et al., 2005; Pillai et al., 2007). These cells may also use glycine for neurotransmission (Goulding, 2009). Although arising from a dorsal progenitor pool, and not being part of the “V” interneurons, the dI6 group of interneurons gives rise to more than one subtype and appears to contribute to motor function (Gross et al., 2002; Muller et al., 2002; Lanuza et al., 2004). These inhibitory neurons are reported in unpublished observations to be commissural and may be involved in right–left alternation, as well (Goulding, 2009). Dmrt3, a novel marker in dl6 interneuron was traced to play a key role in locomotor circuitry and in development of commissural interneurons, and mutation in dmrt3 result in divergent in gait pattern in mice models (Andersson et al., 2012; Vallstedt and Kullander, 2013). Double knockout of *Lhx1* and *Lhx5* results in a loss of *Pax2*, *Viaat*, and *Gad1* expression (Pillai et al., 2007). Furthermore, *Pax2* is required for the maintained expression of *Lhx1, Lhx5, Pax5*, and *Pax8* (Pillai et al., 2007). These cells also express *WT1* (Wilms’ tumor 1; Goulding, 2009). The transcription factor, *Bhlhb5*, marks the dI6, V1 and V2 domains (Ramos et al., 2010). Electrophysiologic characteristics of the dl6 interneurons around a central canal reveal two possible subtypes: one firing trains of action potentials that are loosely coupled to the ventral root output and expressing intrinsic rhythmic activity which suggests a role in locomotor rhythm generation. The other subtype fires action potentials that are tightly coupled to the ventral root output (Dyck et al., 2012).

### dI6 Birth and Early Development

The dI6 neurons are born around E10.5–E11 and originate from a *Pax7*^+^, *Dbx2*^+^, *Ngn1*^+^ and *Ngn2*^+^ pD6 progenitor domain. Post-mitotically they express *Bhlhb5*, *Wt1*, *Lbx1*, *Lhx1*, *Lhx5*, and *Pax2* (references above).

### Late Born Dorsal Interneurons

The dIL neurons represent a second wave of neurogenesis from the dIL progenitor domain that constitutes most of the interneurons in the superficial dorsal horn in Rexed laminae II–IV (Gross et al., 2002; Muller et al., 2002). These cells are formed from common progenitors, and their cell fates are controlled by *Ascl1/Mash1* (**Figure 4M**; Mizuguchi et al., 2006).

### dIL_**A**_ Interneuron Characteristics

The roof-plate independent Class B IL_A_ interneurons are ipsilaterally projecting association neurons that occupy the superficial dorsal horn in Rexed laminae I–III (Gross et al., 2000). These inhibitory neurons are GABAergic and are calbindin+, and a subset express *Gbx1* (John et al., 2005; Mizuguchi et al., 2006).

### dIL_**A**_ Birth and Early Development

The dIL_A_ interneurons are late born (E.13.5) and arise from the dIL progenitor domain that is *Lbx1*^+^, *Lhx1/5*^+^, and *Pax2*^+^(Pillai et al., 2007). The sensory relay interneuron maker *Foxd3* is downregulated in these cells (Gross et al., 2002). *Pax2* has been shown to be necessary for GABAergic differentiation and 98% of these cells also express *Gad1* (*GAD67*; Cheng et al., 2004). The dIL_A_ subclass is dependent on *Ptf1a* and loss of this gene results in trans-fate to the dIL^B^ identity (Glasgow et al., 2005).

### dIL_**B**_ Interneuron Characteristics

The roof-plate independent Class B are *DRG11*^+^ ipsilaterally projecting association interneurons that integrate input from cutaneous sensory neurons that detect noxious stimuli (Chen et al., 2001; Gross et al., 2002). This last-born subclass gives rise to glutamatergic neurons expressing *Tlx1* and *Tlx3* and *Lmx1b* (Cheng et al., 2004; Mizuguchi et al., 2006). The dIL_B_ neurons migrate dorsolaterally to settle in the superficial dorsal horn in Rexed laminae I–III (Gross et al., 2000; Muller et al., 2002). Over 96% of the dorsolateral population of these cells expresses both *Tlx3* and *VGluT2* (Cheng et al., 2004; Glasgow et al., 2005).

### dIL_**B**_ Birth and Early Development

These neurons are born later than the dI1–6 class and express *Lbx1* post-mitotically (Gross et al., 2000; Muller et al., 2002). Mash1 controls the upregulation of Notch signaling to direct formation of dILB from common dIL progenitors (Mizuguchi et al., 2006).

## Discussion

Combinatorial transcriptional control of cell fate is a mature perspective for understanding spinal cord development. This focus on transcription factors has been powerful in two major respects. First, it has allowed the identification of downstream factors that direct cell-specific characteristics, such as neurotransmitter status (Cheng et al., 2004). Second, it has permitted a powerful genetic analysis of spinal neurons, using transcription factors as class-specific tools to drive changes in cell fate and function (Lee et al., 2000; Zhang et al., 2008). Novel techniques are emerging for tracing neuronal circuitry and for the sophisticated manipulation of neuronal activity, including selective cellular ablation, optogenetic activation and silencing, and chemically induced activation and silencing (Boyden et al., 2005; Wickersham et al., 2007; Alexander et al., 2009). These techniques may reach their most exciting potential when coupled with the increasingly specific genetic control available in the spinal cord.

The further refinement of developmental neuronal classes is showing that subclasses may reflect functionally coherent groups of cells that can be mapped onto physiologically identified populations (**Figure 5**). Therefore, as the spinal cord development field grows to incorporate circuitry and behavior, it is merging with the rich field of adult spinal cord electrophysiology that has uncovered major mechanisms of spinal cord function. The combination of these disciplines will advance spinal cord biology to a state that fully encompasses both form and function.

## Conflict of Interest Statement

The authors declare that the research was conducted in the absence of any commercial or financial relationships that could be construed as a potential conflict of interest.
